# Frequency-Dependent Magnetic Susceptibility of Magnetite and Cobalt Ferrite Nanoparticles Embedded in PAA Hydrogel

**DOI:** 10.3390/ijms140510162

**Published:** 2013-05-14

**Authors:** Susanne van Berkum, Joris T. Dee, Albert P. Philipse, Ben H. Erné

**Affiliations:** Van 't Hoff Laboratory for Physical and Colloid Chemistry, Debye Institute for Nanomaterials Science, Utrecht University, Padualaan 8, 3584 CH Utrecht, The Netherlands; E-Mails: s.vanberkum@uu.nl (S.B.); joris.dee@gmail.com (J.T.D.); a.p.philipse@uu.nl (A.P.P.)

**Keywords:** ferrohydrogel, hydrogel, ferrogel, poly(acrylic acid), iron oxide, cobalt ferrite, magnetic nanoparticles, magnetic susceptibility, Brownian relaxation, Néel relaxation

## Abstract

Chemically responsive hydrogels with embedded magnetic nanoparticles are of interest for biosensors that magnetically detect chemical changes. A crucial point is the irreversible linkage of nanoparticles to the hydrogel network, preventing loss of nanoparticles upon repeated swelling and shrinking of the gel. Here, acrylic acid monomers are adsorbed onto ferrite nanoparticles, which subsequently participate in polymerization during synthesis of poly(acrylic acid)-based hydrogels (PAA). To demonstrate the fixation of the nanoparticles to the polymer, our original approach is to measure low-field AC magnetic susceptibility spectra in the 0.1 Hz to 1 MHz range. In the hydrogel, the magnetization dynamics of small iron oxide nanoparticles are comparable to those of the particles dispersed in a liquid, due to fast Néel relaxation inside the particles; this renders the ferrogel useful for chemical sensing at frequencies of several kHz. However, ferrogels holding thermally blocked iron oxide or cobalt ferrite nanoparticles show significant decrease of the magnetic susceptibility resulting from a frozen magnetic structure. This confirms that the nanoparticles are unable to rotate thermally inside the hydrogel, in agreement with their irreversible fixation to the polymer network.

## 1. Introduction

Hydrogels are important for biomedical applications, for instance in drug delivery [1–3] and biosensors [4,5]. They are highly cross-linked networks of polymer strands that can increase their volume by uptake of water without changing their shape or structure [1]. Hydrogels with acidic groups like poly(acrylic acid) (PAA) swell with increasing pH due to increasing repulsive forces of ionized groups [6] and hydrogels may also respond to other factors, such as changes in temperature, ionic strength or other stimuli [1]. This makes hydrogels interesting for biosensor applications. In 2010, van Bruggen and van Zon [7] reported on the feasibility of an *in vivo* biosensor composed of a pH responsive hydrogel containing superparamagnetic nanoparticles on top of a GMR (giant magneto-resistive) sensor. Nanoparticles were magnetically aligned by a high frequency field generated by excitation wires and the field of the nanoparticles was measured by the GMR sensor using a lock-in amplifier. Although the authors were not able to prepare a suitable ferrogel and tested their prototype sensor with a ferrofluid, in principle, the functionality of the sensor is based on the swelling and shrinking of the hydrogel in which the nanoparticles are dispersed. Upon swelling due to an increase of pH, the field of the nanoparticles at the GMR sensor decreases, whereas it increases upon shrinking. In this way, change in pH is indirectly measured by a change in magnetic signal. Here, we investigate the preparation of hydrogels incorporating magnetic nanoparticles and we demonstrate chemical fixation to the polymer network by measuring the frequency-dependent AC magnetic susceptibility.

**Figure 1:**
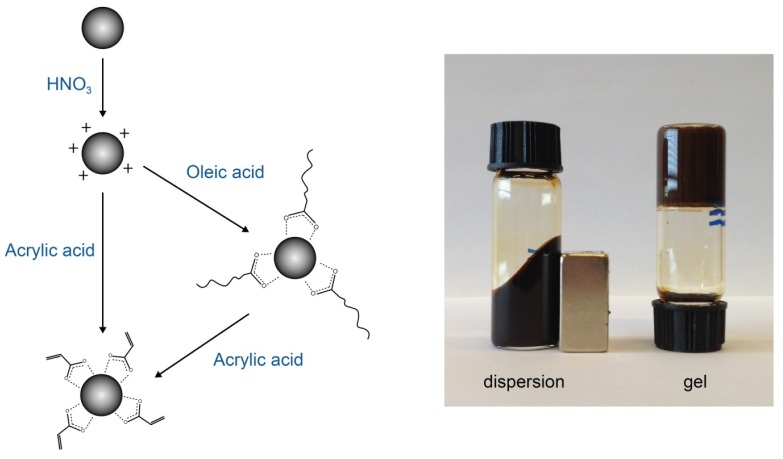
(**left**) Schematic illustration of the surface treatments of our nanoparticles; (**right**) a magnetite (S) dispersion and a hydrogel with incorporated magnetite (S).

The chemical binding of nanoparticles to a hydrogel is achieved by adsorbing polymerizable molecules on the surface of the nanoparticles such that the nanoparticles are not only incorporated in the hydrogel but also become part of the polymer network [8]. Messing *et al.* [9] reported on the transfer of CoFe_2_O_4_ nanoparticles from nonpolar to aqueous media, while at the same time, the surface was functionalized so that the nanoparticles functioned as cross-linkers in a poly(acryl amide) gel. Establishing the function of the nanoparticles as cross-linkers was demonstrated by leakage experiments and by dissolution of the particles upon which the hydrogel fully disintegrated. A similar transfer was described by Vo *et al.* [10] by exchanging oleic acid molecules on TiO_2_ nanoparticles with acrylic acid. In our case, we use adsorbed acrylic acid, to fix nanoparticles to a PAA network. We first positively charge the nanoparticles via a treatment with nitric acid. The first reason for this is to prevent incorporation of large aggregates of particles into the polymer network: positively and negatively charged particles remain well dispersed in aqueous dispersions for long times, but nanoparticles with a negative charge at neutral or high pH immediately form large aggregates upon mixing with the acidic monomer solution, whereas positively charged particles do not. The second reason for giving our nanoparticles initially a positive charge is that spontaneous adsorption of the carboxylic acid group of acrylic acid to the surface of the nanoparticles is facilitated. The end result is that the double bond of the adsorbed acrylic acid points outwards so that it can participate in polymerization, yielding a visually homogeneous gel (Figure 1).

To investigate whether the particles coated with acrylic acid are bound to the hydrogel, our original approach is to examine rotational diffusion by measuring low-field frequency-dependent AC susceptibility spectra in the low-field limit. This technique is known for instance from bio-essays that have been developed to detect magnetically the slowdown of rotational diffusion when molecules absorb to the particle surface, enlarging the hydrodynamic radius [11–15]; it has also been used to monitor the gelation of aqueous gelatin at the scale of magnetic probe nanoparticles [16]. The dimensionless magnetic susceptibility χ gives the magnetization (M) in A/m per applied external field (H) in A/m. When the external magnetic field alternates, the particle magnetic moments of nanoparticles dispersed in a carrier liquid can reorient through two different mechanisms: Brownian relaxation and Néel relaxation [17,18]. Brownian relaxation is the only option for nanoparticles whose magnetic moment is blocked inside the particle: they must physically rotate in the solvent. The characteristic frequency of Brownian relaxation is given by

(1) $$2\pi {\text{f}}_{\text{B}}=\omega_{\text{B}}=2{\text{D}}_{\text{r}}={\text{k}}_{\text{B}}\text{T}/(4\pi \eta {\text{a}}_{\text{h}}^{3})$$

where *f*_B_ is the Brownian relaxation frequency in Hz, ω_B_ the radial frequency in s^−1^, *D*_r_ the rotational diffusion coefficient, *k*_B_*T* the thermal energy, η the viscosity of the solvent, and *a*_h_ the hydrodynamic radius of the nanoparticles. For smaller magnetic nanoparticles, the magnetic moment can rotate fast within the particle, with a characteristic frequency of Néel relaxation given by the following expression,

(2) $$2\pi f_{N}=\omega_{N}=2\pi f_{0}\;\text{exp}[-\frac{KV}{k_{B}T}]$$

where *f*_N_ is the Néel relaxation frequency in Hz, ω_N_ the radial frequency in s^−1^, *f*_0_ the Larmor frequency, *K* the magnetic anisotropy constant, and *V* the magnetic core volume. The frequency dependent magnetic susceptibility has a real and an imaginary part: χ = χ′ – *i*χ″. The real part χ′ has a plateau in the low frequency regime and equals the initial magnetic susceptibility from a magnetization curve without hysteresis (M *versus* H). As the frequency of the alternating field increases, the magnetization of the particles is not able to keep up with the alternating field and the χ′ decreases. The imaginary part χ″ of the magnetic susceptibility shows a maximum at the characteristic relaxation frequency.

**Figure 2:**
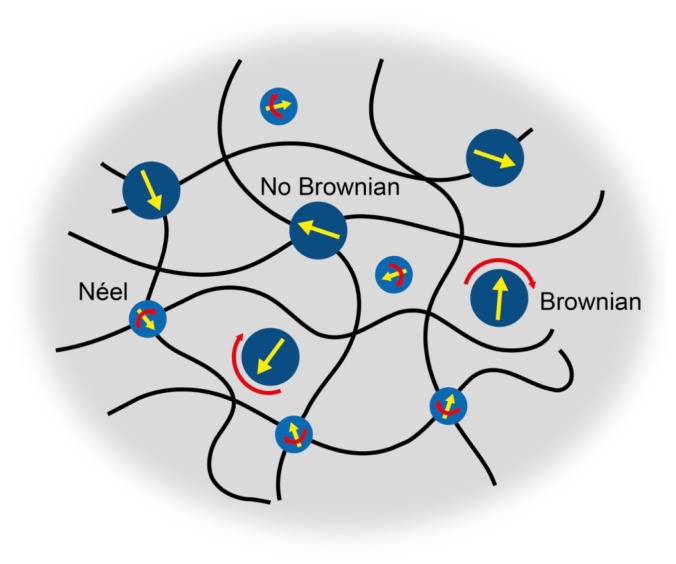
Schematic representation of nanoparticles with different relaxation mechanisms. Particles that are not part of the hydrogel network should be able to rotate whereas particles that are bound to the hydrogel network will not show any magnetic relaxation when the particle magnetic moment is blocked.

The biosensor concept of van Bruggen and van Zon [7] requires a superparamagnetic gel, with magnetic particles that can respond to high operational frequency. Here, we will prepare ferrogels using not only magnetic nanoparticles with fast Néel relaxation, but also with particles having thermally blocked particle magnetic moments. In the latter case, any magnetic susceptibility signal indicates that the particles are physically moving and not well fixed inside the hydrogel (Figure 2). Three types of nanoparticles will be incorporated into gels: superparamagnetic iron oxide nanoparticles (S), larger iron oxide nanoparticles (L) with the same surface chemistry but much longer Néel relaxation, and cobalt ferrite nanoparticles with even longer Néel relaxation due to their high magnetic anisotropy [19].

The following section first describes the magnetic nanoparticles and their surface modification. This is followed by a discussion of fixation of the nanoparticles in the hydrogels as investigated by their frequency-dependent AC magnetic susceptibility.

## 2. Results and Discussion

### 2.1. Characterization of the Magnetic Nanoparticles

**Figure 3:**
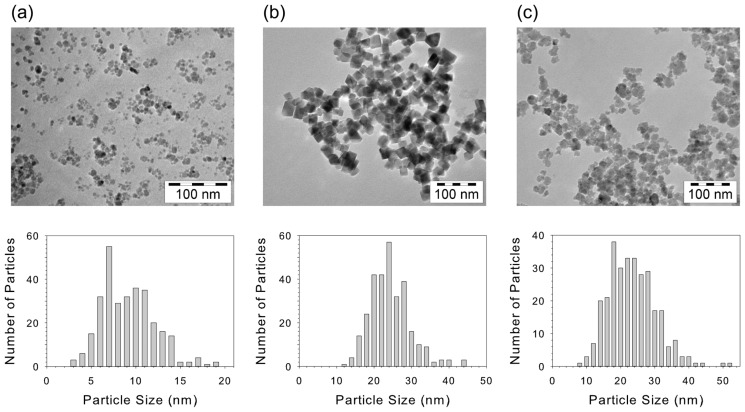
Transmission electron microscopy images of the synthesized nanoparticles with corresponding size distributions: (**a**) magnetite (S), *d* ~ 9 nm, (**b**) magnetite (L), *d* ~ 23 nm, (**c**) cobalt ferrite, *d* ~ 22 nm.

TEM pictures of the three types of synthesized magnetic nanoparticles are shown in Figure 3 together with corresponding size distributions. Due to the aqueous precipitation methods, the size distributions are relatively broad, with polydispersities on the order of 30%. From the black color of the iron oxide synthesis products, it was concluded that they were mainly magnetite as expected from the synthesis methods.

**Figure 4:**
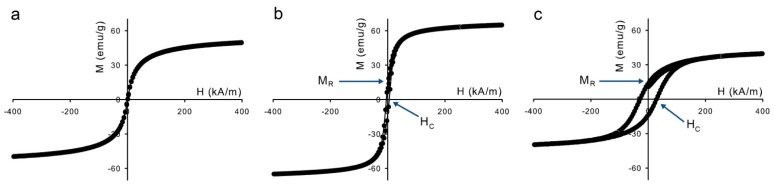
Magnetization curves of the dry nanoparticles at room temperature: (**a**) magnetite (S), (**b**) magnetite (L), and (**c**) cobalt ferrite.

Differences in magnetization dynamics are already visible in the magnetization curves of the dry particles in Figure 4. The slower the magnetic relaxation, the higher the ratio between the remanent magnetization, *M*_R_, and the saturation magnetization, *M*_S_. The magnetic properties are summarized in Table 1. The values for *M*_S_ are lower than those of bulk magnetite, 92 emu/g [20], which is ascribed to nanoparticle surface effects [21–26]. These effects also occur for the cobalt ferrite nanoparticles, whose saturation magnetization is also lower than the bulk value of ~80 emu/g. The magnetization curve in Figure 4a shows that the sample with magnetite (S) has no remanent magnetization, due to rapid Néel relaxation. The magnetite (L) and cobalt ferrite samples, however, show *M*_R_/*M*_S_ values of 0.19 and 0.34 respectively, compared to *M*_R_/*M*_S_ = 0.5 for completely blocked noninteracting nanoparticles with random orientation of the easy axis The last column of Table 1 gives an estimate of the dipolar contact interaction of the nanoparticles,

(3) $$\lambda =\frac{\mu_{0}\mu^{2}}{4\pi \;k_{B}T\;d^{3}}$$

where μ_0_ is the permeability of vacuum, μ is the magnetic dipole moment of a nanoparticle ( $\mu =\frac{\pi d^{3}M_{S}}{6}$), and *d* is the particle diameter.

### 2.2. Surface Modification of Magnetite Nanoparticles Using Acrylic Acid

**Figure 5:**
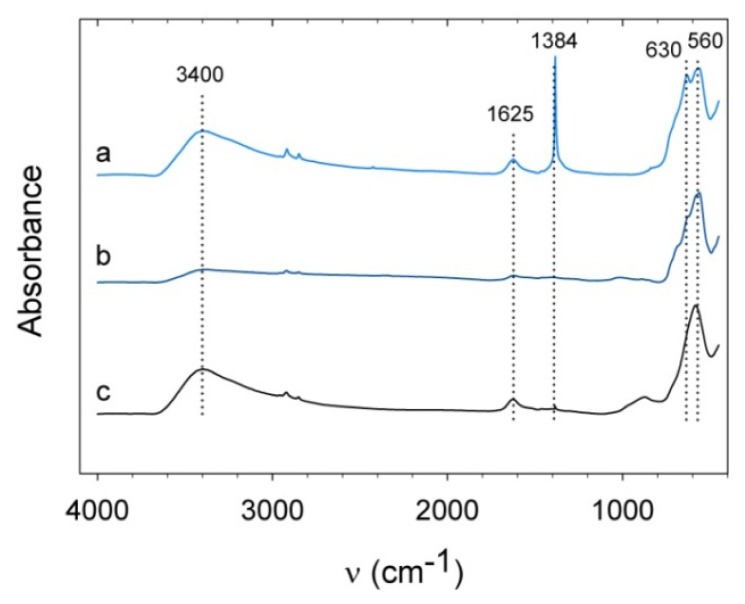
IR spectra of the positively charged nanoparticles: (a) magnetite (S), (b) magnetite (L), and (c) cobalt ferrite.

The infrared spectrum of nitric acid-treated magnetite (S) shows a strong peak of asymmetric stretch of NO_3_^−^ at 1384 cm^−1^ (Figure 5). Magnetite (L) and cobalt ferrite do not show such a strong peak due to their lower surface-to-volume ratio. The spectra of magnetite (S) and cobalt ferrite both show the presence of water by a broad band around 3400 cm^−1^ and a peak at 1600 cm^−1^. Magnetite (S) shows maghemite features at 630 cm^−1^ [27] caused by the heat treatment during KBr sample preparation. Similar features are also observed for magnetite (L) but due to the larger size of the particles, oxidation only affects the surface of the particles and magnetite dominates with its absorbance at 560 cm^−1^. The spectrum for cobalt ferrite displays stretching vibrations of Fe^3+^–O^2−^ in the tetrahedral sites at 574 cm^−1^ [28]. The band around 870 cm^−1^ does not originate from cobalt ferrite [28] or cobalt oxide [29], but we suspect it corresponds to an iron hydroxide phase [30].

**Figure 6:**
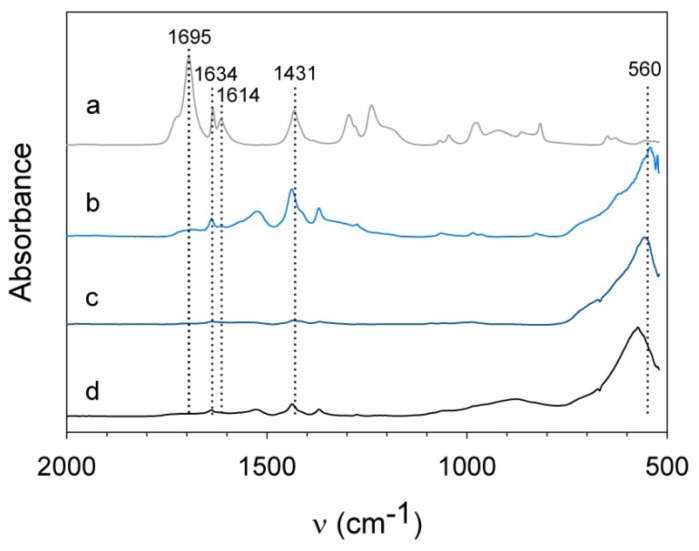
IR spectra of (a) pure acrylic acid and acrylic acid coated nanoparticles: (b) magnetite (S), (c) magnetite (L), and (d) cobalt ferrite.

To fix the nanoparticles to the hydrogel network, surface modification with acrylic acid [10] was performed next. Figure 6 shows the infrared spectra of nanoparticles that were mixed for two minutes with an acrylic acid/water mixture and for comparison the spectrum of acrylic acid. The latter shows characteristic features of acrylic acid: a C=O stretch at 1695 cm^−1^, an C=C stretch at 1634 cm^−1^, an asymmetric stretch of CO_2_^−^ at 1614 cm^−1^, and a symmetric stretch of CO_2_^−^ at 1431 cm^−1^ [31,32]. The peaks between 1200 and 1300 cm^−1^ can be ascribed to C–OH stretch and the weak absorbances between 800 and 1050 cm^−1^ originate from CH out of plane bending [33]. The peak for the C=C stretch at 1634 cm^−1^ slightly shifts to 1639 cm^−1^ when adsorbed onto the nanoparticles surface. The sharp peak at 1695 cm^−1^ disappears for acrylic acid adsorbed onto nanoparticles, whereas the symmetric stretch of CO_2_^−^ is enhanced and shifts to 1438 cm^−1^, pertaining to an adsorbed carboxylate group [32]. This is valid for both magnetite (S) and cobalt ferrite. The surface group bands for magnetite (L) are much weaker due to the lower surface-to-volume ratio of the larger particles.

**Figure 7:**
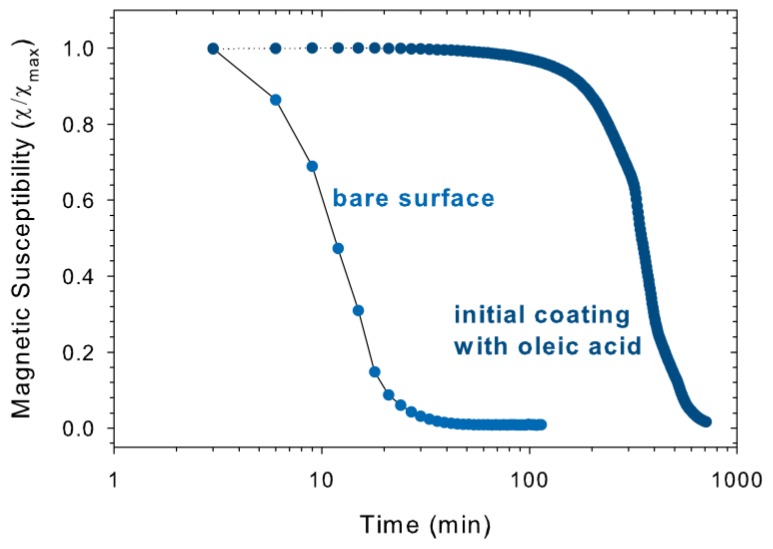
Low-field magnetic susceptibility (real susceptibility at 1 Hz) of magnetite (S) with a positively charged bare surface and oleic acid coated magnetite (S) dispersed in 90 vol% solution of acrylic acid in water, normalized to their initial value χ_max_ at 1 Hz. Positively charged magnetite (S) shows an immediate decrease of magnetic susceptibility due to dissolution of the particles, whereas the decrease of the magnetic susceptibility of oleic acid coated nanoparticles is slowed down by ligand exchange.

We discovered that treatment time of the acrylic acid surface modification method must be kept limited to prevent dissolution of the particles, especially when they are not initially covered with other adsorbed molecules. When the procedure was performed with bare particles overnight as was done with oleic acid coated particles by Vo *et al.* [10], the color of the dispersion changed orange and the magnetic susceptibility decreased dramatically. Magnetic susceptibility measurements at 1 Hz show that the magnetic susceptibility for positively charged iron oxide nanoparticles decreased to the noise level within 40 min (Figure 7). Oleic acid coated nanoparticles, however, required 10 h for a similar decrease, indicating that dissolution is slowed down because ligand exchange must occur. Figure 7 demonstrates that spontaneous adsorption of acrylic acid onto the surface of iron oxide NPs is rapid; therefore mixing of positively charged iron oxide NPs with the monomer mixture for two minutes should be sufficient for adsorption of acrylic acid.

### 2.3. Magnetic Relaxation of Ferrogels

**Figure 8:**
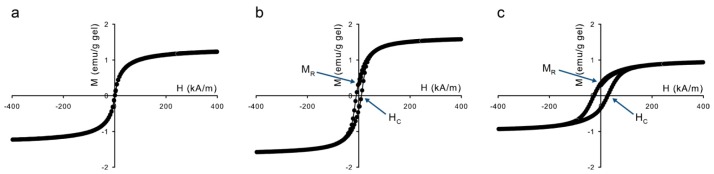
Magnetization curves of (**a**) magnetite (S), (**b**) magnetite (L), and (**c**) cobalt ferrite nanoparticles embedded in a hydrogel.

**Figure 9:**
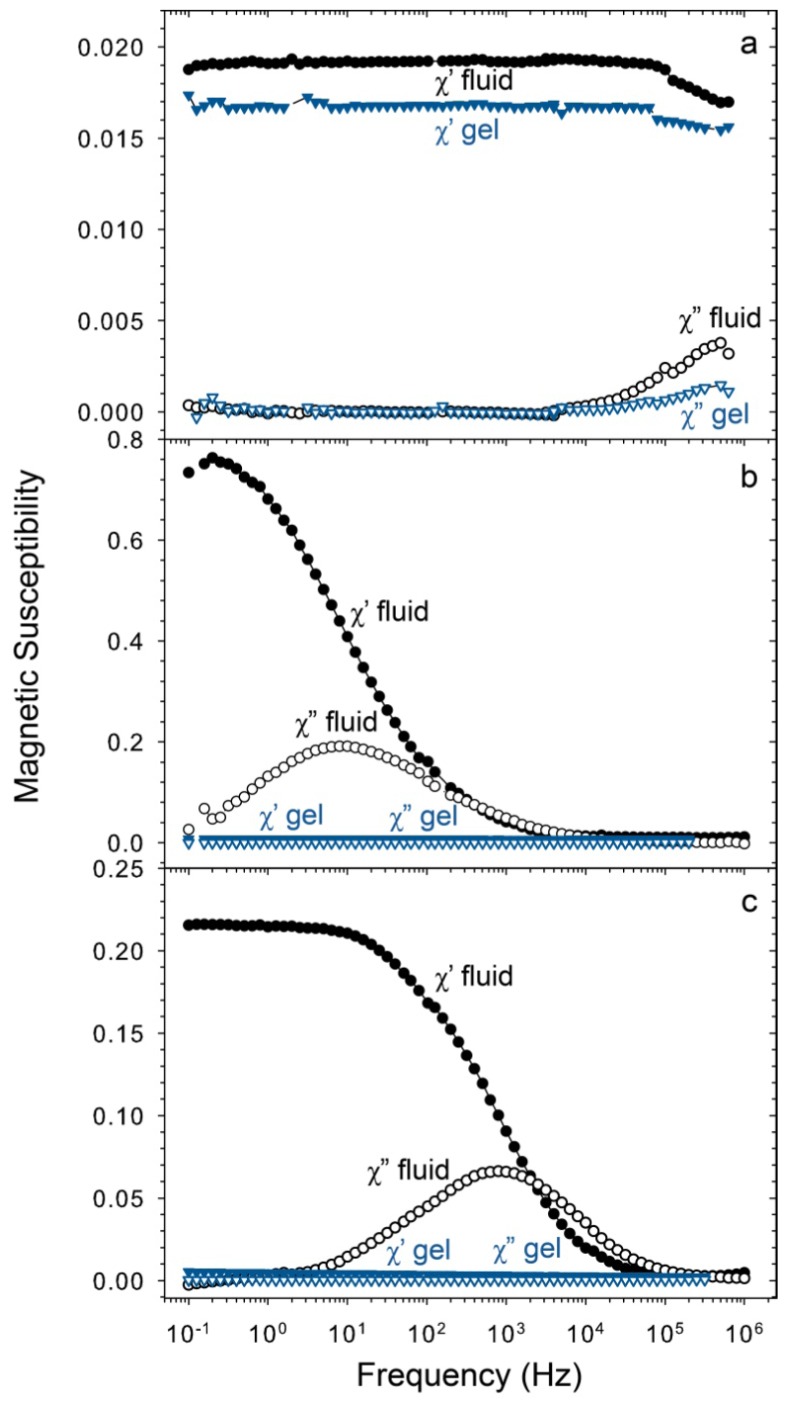
Frequency-dependent AC susceptibility spectra of liquid dispersions (“liquid”) and poly(acrylic acid) hydrogels (“gel”) incorporating: (**a**) 1.6 wt% of magnetite (S) nanoparticles, (**b**) 2 wt% of magnetite (L) nanoparticles, and (**c**) 2 wt% of cobalt ferrite nanoparticles. After immobilization of the nanoparticles in a gel, the rapid Néel relaxation of the small particles continues (**a**), except for a small fraction of larger particles. The magnetic relaxation of the larger magnetite particles (**b**) and cobalt ferrite (**c**) is almost fully quenched after incorporation in hydrogel because no Néel relaxation occurs and the particles are immobilized inside the polymer network.

For the hydrogels with embedded nanoparticles, magnetization curves are shown in Figure 8. The magnetic properties are summarized in Table 2. The saturation magnetization of each sample is in the same order as expected from the volume fraction of magnetic material present. Striking is that the relative remanence *M*_R_/*M*_S_ and coercive field of magnetite (L) are increased by 27% and 95% respectively compared to the values in Table 1. This effect is ascribed to changes in the magnetic interactions between the nanoparticles within the gel. Dipolar structures are formed when the contact interaction of magnetic nanoparticles is sufficiently high compared to the thermal energy k_B_T [34,35] which is the case for magnetite (L) and cobalt ferrite, see Table 1, where the λ is the highest for magnetite (L) due to its large particle size. Introducing magnetic interactions locks the orientation of the magnetic dipoles inside the structures, which slows down the magnetization dynamics [36]. Evidence for particle interactions is given by the characteristic relaxation frequency of magnetite (L) and cobalt ferrite in dispersion shown in Figure 9, which reveals the presence of clusters, confirming that the particles are close enough for particle-particle interaction. For cobalt ferrite nanoparticles, a similar increase in *M*_R_/*M*_S_ is observed, although the coercive field has decreased slightly.

Figure 9 shows magnetic susceptibility spectra for all three types of magnetic nanoparticles used in this study. The nanoparticles were measured both in dispersion and in hydrogel to investigate the difference in magnetic susceptibility between bound particles and particles that have a positively charged surface and are free to rotate in dispersion.

The characteristic relaxation frequency for magnetite (S) differs when the particles are free in dispersion or embedded in a hydrogel. This can be explained by the wide size distribution and the effective relaxation time, τ_eff_,

$$\frac{1}{\tau_{eff}}=\frac{1}{\tau_{N}}+\frac{1}{\tau_{B}}$$

where *τ*_N_ is the Néel relaxation time and τ_B_ is the Brownian relaxation time. The magnetic moments of the particles in dispersion are all free to rotate by a combination of both mechanisms, whereas this is not the case for particles in a hydrogel, for which Néel relaxation is the only remaining option. This is clearly the case in Figure 9a: in liquid dispersion, the magnetic moments of the particles are all free to rotate and τ_eff_ results from both relaxation mechanisms, whereas in the hydrogel network, Brownian relaxation is excluded; about 10% of the magnetic moments can no longer rotate since the particles are so large that their Néel relaxation is too slow. This leads to a shift in the characteristic relaxation frequency, since the effective relaxation time is now a result of only the small particles with fast Néel relaxation.

The imaginary part of the spectrum of the magnetite (L) nanoparticles in dispersion has a maximum at 8 Hz in Figure 9b, corresponding to clusters of about 400 nm. This is in good agreement with the observation that the sample showed sediment after one day. The characteristic relaxation frequency of the cobalt ferrite nanoparticles is 800 Hz in liquid dispersion in Figure 9c, corresponding to clusters of about 80 nm. In a carrier liquid the thermally blocked magnetite (L) and cobalt ferrite nanoparticles will relax by the Brownian mechanism [17,34–36], resulting in a strong decrease in magnetic susceptibility signal for nanoparticles immobilized in the hydrogel network, comparable to what happens when a ferrofluid is frozen [37]. The large decrease in magnetic susceptibility of the nanoparticles when incorporated in hydrogel means that the particles are no longer able to rotate.

The irreversible fixation of the nanoparticles to the polymer network was expected from the followed chemical methods, where the nanoparticles acted as cross-linkers (Figure 1). Contrary to [8,9] the nanoparticles in our case were not the sole cross-linkers, but by measuring the magnetic susceptibility, we could single out the motion of the nanoparticles and conclude that they were fixed in the gels. No leakage of nanoparticles was observed by us upon swelling and shrinking of the ferrogels, in contrast to studies on ferrogels in which part of the particles was still free to rotate [38,39]. For application in biosensors of the type described by van Bruggen en van Zon [7], our ferrogel with small magnetite particles (S) seems highly suitable: the particles are fixed to the polymer network and they respond strongly to alternating magnetic fields, at least up to an operational frequency of 50 kHz.

## 3. Experimental Section

### 3.1. Materials

Iron (II) chloride tetrahydrate (FeCl_2_·4H_2_O, >98%), iron (II) chloride hexahydrate (FeCl_3_·6H_2_O, 97%), and iron sulfate heptahydrate (FeSO_4_·7H_2_O, 99%) were purchased from Sigma. Acrylic acid (AA, 99% anhydrous), diethyleneglycol diacrylate (DEGDA, 75%), and oleic acid (OA, techn. 90%) were obtained from Aldrich. Cobalt (II) chloride hexahydrate (CoCl_2_·6H_2_O, >98%) was purchased from Fluka. Ethanol (EtOH, *p.a.*), *n*-hexane (*p.a.*), potassium nitrate (KNO_3_, 99%), and sodium hydroxide (NaOH, 99%) were purchased from Merck (Darmstadt, Germany). Ammonia solution (NH_4_OH, 28wt%–30wt% in water), sulfuric acid (H_2_SO_4_, 96%), hydroxylethyl acrylate (HEA, 97% stabilized), and 2,2′-azobis(2-methylpropionamidine) dihydrochloride (V-50, 98%) were obtained from Acros Organics (Geel, Belgium). Toluene (techn.) was purchased from Interchema. All chemicals were used as received. Deionized water purified by a Millipore purification system (MILLIPORE Synergy 185) was used in all syntheses and washing steps.

### 3.2. Preparation of Magnetic Nanoparticles

Magnetite (S) nanoparticles—Small magnetite nanoparticles were prepared following the method reported by Massart *et al.* [40]: 3.93 g (20 mmol) FeCl_2_·4H_2_O was dissolved in 10 mL of 2 M HCl and added to 10.89 g (40 mmol) FeCl_3_·6H_2_O dissolved in 40 mL of water. The mixed solution was added rapidly to 500 mL of 0.7 M ammonia solution under vigorous stirring to form a black precipitate. The mixture was stirred vigorously for another 25 min and subsequently sonicated for 5 min. The particles were washed three times by magnetic sedimentation and redispersion in water. The particles were finally redispersed in water.

Magnetite (L) nanoparticles—Large magnetite nanoparticles were prepared as described by Andres Vergés *et al.* [41]: 0.56 g NaOH (0.072 M) and 1.05 g KNO_3_ (0.052 M) were dissolved in 80 mL of water and 100 mL of ethanol, forming solution A. For solution B, 56.4 μL of concentrated H_2_SO_4_ was diluted in 100 mL of water. From this dilution, 20 mL was used to dissolve 1.39 g FeSO_4_·7H_2_O (0.025 M). Both solutions were degassed with N_2_ for 2 h, while solution A was also stirred. After degassing, solution B was quickly added to solution A while stirring vigorously. After 10 min, stirring was stopped and the flask was placed in an oil bath that was preheated to 90 °C and left for 24 h under nitrogen. After heating, the oil bath was removed and the flask was left to cool down to room temperature. The nanoparticles were separated by magnetic decanting and washed until a stable dispersion was obtained, typically after 7 washing steps. The nanoparticles were finally dispersed in water.

Cobalt ferrite nanoparticles—The cobalt ferrite nanoparticles were prepared by the method reported by Tourinho *et al.* [42]: 2.39 g (10 mmol) CoCl_2_·6H_2_O was dissolved in 5 mL of 2 M HCl and added to 5.48 g FeCl_3_·6H_2_O (20 mmol) dissolved in 40 mL of water. This mixture was briefly stirred and added to a boiling solution of 1 M NaOH (200 mL) under vigorous stirring. Immediately a black/brown precipitate was formed and the mixture was heated to reflux for 1 h. When cooled down to room temperature, the particles were magnetically sedimented and washed three times with 70 mL of water. The particles were redispersed in 50 mL of water and 30 mL of 2 M HNO_3_ was added. The mixture was vigorously stirred and 30 mL of 0.35 M Fe(NO_3_)_3_·9H_2_O was added. The mixture was heated to reflux for 1 h and cooled down to room temperature. The particles were magnetically sedimented, washed once with water, and subsequently redispersed in Millipore water.

### 3.3. Positive Charging of Magnetic Nanoparticles

For nanoparticle fixation to the hydrogel network, we found that the particles must be positively charged to facilitate spontaneous adsorption of acrylic acid. For this, approximately 200 mg of nanoparticles of the original dispersion was taken. The particles were diluted with 500 mL of 2 M HNO_3_ and stirred for 30 min. Subsequently, the nanoparticles were washed several times until a stable dispersion in water was obtained. For magnetite (L) also ultrasonication was used to break up large aggregates present in the dispersion.

### 3.4. Surface Modification of Magnetic Nanoparticles with Oleic Acid

For some dissolution experiments, magnetite (S) nanoparticles were coated with oleic acid. In a typical experiment, approximately 200 mg of charge stabilized magnetite nanoparticles were diluted with 250 mL water. This dispersion was stirred vigorously while oleic acid was added. When stirring was stopped, oleic acid formed a thick liquid phase on top of the water phase. If the water phase still contained nanoparticles, more oleic acid was added and the mixture was stirred again. This process was repeated until the water phase was white and turbid, typically after addition of 15 mL of oleic acid. The black top phase was washed with ethanol using a separation funnel, and the particles were finally dispersed in toluene or hexane.

### 3.5. Preparation of Hydrogel and Fluid Samples for Magnetic Susceptibility Measurements

For magnetic susceptibility measurements, two types of samples were used: a liquid dispersion of magnetic nanoparticles and magnetic nanoparticles incorporated in a hydrogel. For both types of sample, an equal amount of magnetic material was used. As initiator, V-50 was selected as there was no visible aggregation of the nanoparticles upon mixing with the monomers, in contrast to preliminary experiments with free radical polymerization using ammonium persulfate and *N*,*N*,*N*′,*N*′-tetramethylethylenediamine (TEMED). The monomer mixture composition was as follows: 0.33 mL of acrylic acid, 5.00 mL of hydroxyethyl acrylate, and 0.59 mL of diethylene glycol diacrylate. For the magnetite (S) dispersion sample, 0.8 mL of 20 mg/mL dispersion was diluted with 0.2 mL of water to obtain a 16 mg/mL dispersion. For the hydrogel sample, 0.8 mL of 20 mg/mL dispersion was diluted with 0.05 mL of water, 0.1 mL of monomer mixture, and 0.05 mL of initiator solution (V-50, 12 mg/mL). The mixture was shaken and transferred to a measurement tube. The tube was put in an oven at 70 °C for polymerization for 15 min. The sample preparation and total sample volume were similar for magnetite (L) and cobalt ferrite. For magnetite (L), 0.63 mL of a 32 mg/mL stock dispersion was used and for cobalt ferrite 0.5 mL of a 40 mg/mL stock dispersion was used.

### 3.6. Characterization

Transmission electron microscopy images were obtained from a Philips Tecnai 12 microscope operating at 120 kV. The particle size was determined by measuring at least 300 particles individually by hand using iTEM software (Olympus, Münster, Germany).

Infrared spectra were recorded on a Frontier FT-NIR/MIR Spectrometer from Perkin Elmer. Samples of positively charged particles for IR were prepared by adding a small amount of dispersion to 250 mg KBr, with approximately 1 mg of magnetic nanoparticles. The KBr samples were dried overnight at 80 °C, ground to fine powder and pressed into pellets. Before measuring, the pellets were dried for 4 h at 80 °C to remove water that was incorporated during preparation of the pellets. Samples of particles coated with acrylic acid were prepared by exposing the particles to a 1:1 mixture of acrylic acid and water for a few minutes. The particles were magnetically sedimented and washed three times with acetone. The particles dispersed in acetone were put on an ATR crystal and a measurement could be done when all acetone had evaporated.

Magnetization curves were measured at room temperature on a Micromag 2900 alternating gradient magnetometer from Princeton Measurement Corporation.

Frequency dependent magnetic susceptibility measurements were performed at 22 °C in the low-field limit (*H* < 170 A/m) on a homebuilt setup described by Kuipers *et al.* [43,44], whose 0.1 Hz–1 kHz frequency range was extended to 1 MHz using 7280 DSP lock-in amplifiers.

## 4. Conclusions

Hydrogels were prepared in which magnetic nanoparticles act effectively as cross-linkers. To examine the rotational mobility of the particles, our approach was to measure the low-field AC magnetic susceptibility in a frequency range that corresponds to Brownian rotation of single nanoparticles. The large decrease of the AC susceptibility of hydrogels with cobalt ferrite compared to the dispersion is due to its high magnetic anisotropy and the prohibition of Brownian relaxation in the hydrogel. The same is observed for iron oxide particles with a diameter of 23 nm, whose magnetic particle moment is thermally blocked. Smaller iron oxide particles with a diameter around 9 nm exhibit fast Néel relaxation at frequencies of 1 MHz and below, so that it does not matter whether the nanoparticles are fixed inside a hydrogel or dispersed in a liquid. However, the chemistry of the small iron oxide particles is the same as that of the larger ones, and we conclude that they also were irreversibly bound to the polymer network. This was expected *a priori* from our use of nanoparticles with adsorbed polymerizable surface species but now has been demonstrated by characterizing the rotational diffusion of the larger particles. A ferrohydrogel with frequency-independent response up to at least 1 MHz appears suitable for *in vivo* biosensors of the type proposed by van Bruggen and van Zon, which was previously tested at 50 kHz [7]. Currently, we are carrying out a systematic study of the swelling of our ferrohydrogels and how this can be detected magnetically.

## Figures and Tables

**Table 1:** Magnetic properties of the nanoparticles. The standard deviations given in parentheses are from performing triplicate analyses.

	*M*_S_ (emu/g)	*M*_R_/*M*_S_	*H*_C_ (kA/m)	λ
Magnetite (S)	54.8 (1.2)	0.007 (0.001)	-	0.4
Magnetite (L)	67.5 (0.3)	0.187 (0.003)	5.09 (0.05)	10
Cobalt ferrite	38 (4)	0.343 (0.013)	34 (4)	3

**Table 2:** Magnetic properties of the ferrogels. The standard deviations are given in parentheses from performing triplicate analyses.

	*M*_S_ (emu per g gel)	*M*_R_/M_S_	*H*_C_ (kA/m)
Magnetite (S)	1.20 (0.06)	0.009 (0.000)	-
Magnetite (L)	1.63 (0.04)	0.238 (0.006)	9.91 (0.15)
Cobalt ferrite	0.95 (0.10)	0.375 (0.012)	26.8 (0.4)
